# Risk of violence in drug rehabilitation centers: perceptions of people who inject drugs in Tijuana, Mexico

**DOI:** 10.1186/s13011-015-0044-z

**Published:** 2016-01-26

**Authors:** Alicia Yolanda Harvey-Vera, Patricia González-Zúñiga, Adriana Carolina Vargas-Ojeda, Maria Elena Medina-Mora, Carlos Leonardo Magis-Rodríguez, Karla Wagner, Steffanie Anne Strathdee, Daniel Werb

**Affiliations:** Division of Global Public Health, University of California, San Diego, School of Medicine, San Diego, California USA; Universidad Autónoma de Baja California, Facultad de Medicina y Psicología, Tijuana, Baja California Mexico; Instituto Nacional de Psiquiatría de México, Ciudad de Mexico, Distrito Federal Mexico; Centro Nacional para la Prevención y Control del VIH/SIDA, Ciudad de Mexico, Distrito Federal Mexico

**Keywords:** HIV testing, IDUs, PWID, Mexico, Drug rehabilitation, Fear, Violence

## Abstract

**Background:**

In 2009, Mexico reformed its health law to partially decriminalize drug possession considered for personal use and to increase mandatory referrals to certified drug rehabilitation centers in lieu of incarceration. Concurrently, news media reported violent attacks perpetrated by drug cartels against Mexican drug rehabilitation centers and instances of human rights violations by staff against people who inject drugs (PWID) in treatment. In many cases, these violent situations took place at “Peer Support” (Ayuda Mutua) drug rehabilitation centers that house a large number of drug-dependent PWID. In an effort to understand barriers to treatment uptake, we examined prevalence and correlates of perceived risk of violence at drug rehabilitation centers among PWID in Tijuana, Mexico.

**Methods:**

Secondary analysis of baseline data collected between March 2011 and May 2013 of PWID recruited into a prospective cohort study in Tijuana. Interviewer-administered surveys measured perceived risk of violence at drug rehabilitation centers by asking participants to indicate their level of agreement with the statement “*going to rehabilitation puts me at risk of violence*”. Logistic regression was used to examine factors associated with perceived risk of violence.

**Results:**

Of 733 PWID, 34.5 % perceived risk of violence at drug rehabilitation centers. In multivariate analysis, reporting ever having used crystal methamphetamine and cocaine (separately), having a great or urgent need to get help for drug use, and ever receiving professional help for drug/alcohol use were negatively associated with perceived risk of violence at drug rehabilitation centers, while having been told by law enforcement that drug rehabilitation attendance is mandatory was positively associated with perceived risk of violence. All associations were significant at a 0.05 alpha level.

**Conclusion:**

The perception of violence at drug rehabilitation centers among PWID does not represent the lived experience of those PWID who attended professionalized services, reported a great or urgent need to get help for their drug use and had a history of using crystal and cocaine. Professionalizing service delivery and engaging law enforcement in their new role of decriminalization and service referral for PWID could address the perceptions of violence at drug rehabilitation centers. Similarly, health authorities should expand periodic inspections at drug rehabilitation centers to guarantee quality service provision and minimize PWIDs’ concerns about violence.

**Electronic supplementary material:**

The online version of this article (doi:10.1186/s13011-015-0044-z) contains supplementary material, which is available to authorized users.

## Background

Drug rehabilitation is a complex process which can include a diversity of treatment modalities with relative levels of effectiveness [1, 2]. The World Health Organization (WHO) has noted that maximizing the proportion of drug-dependent individuals receiving effective addiction treatment would benefit both those individuals as well as society at large [1]. The WHO further states that this can be achieved by guaranteeing that quality drug rehabilitation is available, accessible and affordable [1]. The United Nations Office on Drugs and Crime (UNODC) has also suggested that rates of enrolment in addiction treatment may be increased by improving the interaction between the health-care system and the criminal justice system [2]. In turn, successful drug rehabilitation reduces the level of drug-related harms experienced by communities and is a more cost-effective approach to controlling drug addiction compared with criminal justice involvement [2]. However, the content, features, and delivery of a drug rehabilitation program are important in determining treatment outcomes [1, 2]. In order to promote a consistent level of treatment effectiveness, the National Registry of Evidence-based Programs and Practices (NREPP), a program of the Substance Abuse and Mental Health Service in the US has compiled more than 300 evidence-based interventions that have been proven effective at drug rehabilitation, including motivational interviewing [3] and relapse prevention models such as The Matrix [4].

Unfortunately, instances of basic human rights abuses such as detoxification without medication, hard labor, physical and psychological abuse and withholding of food as punishment for noncompliance have been documented at drug rehabilitation centers worldwide, particularly in resource limited countries such as Myanmar, Cambodia, China, Thailand, and Vietnam [5–8]. Among potential human rights abuses, exposure to violence and mistreatment in drug treatment centers has been shown to reduce the effectiveness of addiction treatment [8–10].

Previous research has documented a relationship between drug use, drug dealing and violence [11, 12]. Some authors have found that the type of drug used influences whether the person is the perpetrator or victim of violence [13]. In some countries, law enforcement violence against drug users has been routine and severe [14–16]. Other authors have shown how violence can shape the environment in which a variety of drug-use related harms occur [14–16]. Violence or the threat of violence can also contribute to the social and economic marginalization of drug users and increase the potential of unintended public health harms, such as increased risk of HIV and other sexually transmitted infections (STIs) or blood-borne pathogens such as Hepatitis B or C [14, 16–18].

In the particular case of the drug rehabilitation setting, we define violence as the use of physical force or power, threatened or actual, against oneself, or other individuals, group or community that results in the likelihood of resulting in injury, death, or psychological harm among others [19, 20]. Sources of violence for PWID in Tijuana may include interactions with law enforcement, the actions of local drug cartels against drug treatment centers, ill-conceived drug rehabilitation therapy that includes practices reported in Mexico and other settings such as forcing clients to stand on a single cinder block for hours; prolonged kneeling on beans, rice or used bottlecaps, holding weights with extended arms, and being deprived of sleep and food; or the actions of rogue service providers or patients themselves within the facilities [18, 21–25].

### Mexico’s addiction treatment landscape

In Mexico, historically, peer-support (i.e., ‘ayuda mutua’) drug rehabilitation programs were developed in response to the scarcity of professional services or resources to address the rehabilitation needs of low-income drug and alcohol-dependent people [21, 25–31]. The current peer support (ayuda mutua) programs share some elements of the traditional 12 Step services, but have evolved and expanded services (i.e., in-patient addiction treatment) [21, 28, 30, 31]. In the 1980s, Annex (“Anexos”, in Spanish) facilities were created to offer room and board for those PWUD who did not have a place to stay. In exchange for these services, clients helped with chores, such as cleaning, cooking and running errands, until they were functionally able to return to the community [25, 28, 29]. Similarly, “rehabilitation farms” (“granjas de rehabilitacion”) were developed exclusively for male drug users to stay for a period of 1 year working on managing their drug dependence [26]. These programs typically do not offer opioid substitution therapy and do not always provide treatment for withdrawal symptoms. Some authors such as Marin-Navarrete have proposed naming these two types of peer support programs (ayuda mutua and granjas de rehabilitacion) as Mutual-Aid Residential Addiction Care Centers (Centros Residenciales de Ayuda-Mutua para la Atención de las Adicciones ‘CRAMAA’) [21, 30–32]. It is estimated that some 700,000 have been attending this type of peer support programs [32, 33].

The quality of care at drug rehabilitation centers in México is regulated by the National Policy for the Prevention, Treatment, and Control of Addictions, which was updated in 2009 [33, 34]. The federal legislation clearly outlines the standards required of each treatment modality, as well as patients’ rights during their stay in rehabilitation, and provides guidelines for both out-patient and in-patient treatment centers.

### Violence in Mexico’s drug rehabilitation centers

Several authors have reported on consumer satisfaction with treatment services provided [17, 18, 21, 25, 28, 32] and instances of mistreatment at addiction treatment centers in Tijuana have also been covered by news media in recent years [22–25, 35]. In particular, evidence suggests that physical abuse and violence have taken place at peer support-based centers, in some cases culminating in legal action against the programs [25–27]. For instance, Syvertsen et al. [18] observed that, among PWID in Tijuana in 2005, 22 % of the sample (*n* = 222) reported instances of abuse during drug treatment rehabilitation experiences. Among a subset of participants describing mistreatment at drug rehabilitation centers (*n* = 25), 72 % reported physical abuse and 52 % reported verbal abuse. Marin-Navarrete et al. [32] and Lozano-Verduzco et al., [21] also describe instances of sexual, physical and psychological violence and verbal abuse among men during their stay in peer support (ayuda mutua) centers in Mexico City. The authors conclude that providers at these programs justified the use of violence as a means to achieve humility and subordination, in their misinformed belief that they are keys to successful substance use rehabilitation [21, 32].

### Mexico’s drug law reform

Mexico experienced a dramatic escalation of drug-related violence, from 2826 to 15,273 drug-related deaths in 2007 and 2010, respectively [35–37]. In response, the government set out a nationwide strategy to counteract the effects of public insecurity, with a major emphasis on increasing the uptake and availability of addiction treatment for drug-dependent individuals. As part of this strategy, in 2009 a landmark federal legislation regulating the production, consumption, distribution and trafficking of illicit substances was passed [38]. Importantly, the new legislation drew a legal distinction between people who use drugs recreationally, drug-dependent individuals, and drug dealers. Under the law reform, individuals stopped by law enforcement and found to be in possession of quantities of drugs (e.g., heroin, cocaine, methamphetamine, and marijuana, among others) under a legal threshold for personal use are referred to drug rehabilitation centers, and are sent to mandatory drug rehabilitation upon their third infraction [38]. Other aspects of the reform call for the introduction of the concept of harm reduction into federal drug laws and the re-structuring and scale-up of the drug rehabilitation system to accommodate increased demand. Federal and state institutions were given until August 2012 to develop the necessary mechanisms to meet the requirements of the revisions [38].

Given reports of violence at drug rehabilitation centers, one would expect that compared to other drug users, PWID would be more likely to experience it due to the severity of their drug use and their tendency to be immersed in street culture where violence is highly prevalent [14, 39].

### Conceptual model

The ecological framework considers that a multitude of factors at four levels – individual, interpersonal, community and societal – can explain why some people or groups have a greater perception of risk of violence, while others are protected from it [14–16, 40]. At the individual level, personal history and biological factors influence how people behave and their perception of becoming a victim or perpetrator of violence; interpersonal relationships with family members, friends, and peers may also influence the risk of having a perception of becoming a victim or perpetrator of violence; community and societal contexts broadly dictate the nature of social relationships and thereby influence an individual’s perception of violence. Furthermore, these factors at the individual, interpersonal, community and societal factors separately or in combination can facilitate or impede the access to drug rehabilitation services among injection drug users [14–16]. Attitudes and perceptions related to violence (such as denial, stigma, discrimination, fear, threats experienced or witnessed) can also play a significant role in individuals’ decision to seek help for their drug use [14–16]. In our study, we focus on interpersonal violence at a community level as we study the perception of PWID regarding drug treatment centers in Tijuana, Mexico.

## Methods

### Study design

We employed STrengthening the Reporting of OBservational studies in Epidemiology (STROBE) guidelines for this study [41]. Please refer to Additional file 1 - STROBE Statement—checklist of items that should be included in reports of observational studies [41]. We used cross-sectional data collected from a convenience sample of 733 PWID who completed baseline assessments for Proyecto El Cuete Phase IV between March 2011 and May 2013 [42].

### Setting and participants

This study involves Proyecto El Cuete Phase IV participants (*N* = 733) who underwent baseline interviewer-administered surveys and indicated their level of agreement with the statement “going to rehabilitation puts me at risk of violence”. Proyecto El Cuete Phase IV is an ongoing prospective cohort study of PWID in Tijuana, Mexico [42]. To be eligible, individuals had to report injecting illicit drugs in the past month (confirmed by visual assessment of ‘track marks’); residing in Tijuana; speaking Spanish or English; and being at least 18 years of age. Participants complete interviewer-administered surveys at baseline and follow-up visits scheduled every 6 months. The surveys elicit data on sociodemographic factors; drug use history; sexual activity; access to, experiences with, and attitudes towards drug treatment services; interactions with law enforcement and violence, and other relevant domains. At each visit, participants also undergo rapid HIV testing and are provided with pre- and post-test counseling. Participants requiring medical attention are referred to municipal clinics where they can receive free care under Mexico’s universal health care system.

### Measures

The dependent variable for this analysis was a dichotomous measure assessing participants’ perceived risk of violence at drug rehabilitation centers. Participants were asked to indicate their level of agreement with the following statement: “*going to rehabilitation puts me at risk of violence*.” Responses were measured using a 6-point Likert scale (1 = Strongly Disagree, 2 = Disagree’ 3 = A little disagree, 4 = A little agree, 5 = Agree and 6 = Strongly Agree) that was transformed into a categorical variable for the purposes of this analysis (1 = Agree (grouped response items 4–6) vs. 0 = Disagree (grouped response items 1–3). Independent variables capture information at the individual, contextual and structural-level and are described below.

*Individual level variables* involved both sociodemographic and drug-related variables. Specifically, sociodemographic variables included: age (in years), gender (female vs. male), years of education completed, main source of income in the past year (no income,formal source of income [i.e., a legal job with pay], vs. informal source of income [i.e., informal work, selling drugs, running a shooting gallery, sex work, etc.]), homelessness in the past 6 months (defined as sleeping or living in a car, abandoned building or on the streets vs. living or sleeping at other places), and number of years living in Tijuana. Drug-related variables included: age of first drug use (in years), age of first drug injection (in years), and lifetime use of cocaine, heroin, or crystal methamphetamine (no lifetime use of any drugs vs. lifetime use of at least one drug).

Additionally, participants were asked “To what extent would you say that you currently need help for your drug use?” (0 = no need, 1 = some need, 2 = great need, to 3 = urgent need).

*Interpersonal-level variables* included: ever having been stopped by law enforcement (for any offense or suspicion of committing an offense), and ever having been told by law enforcement or other officials to attend mandatory drug rehabilitation.

*Community and societal level variables* included those associated with drug rehabilitation services such as: ever receiving professional help for drug or alcohol use, the number of times that individuals reported receiving professional help for drug use, ever being enrolled in a drug rehabilitation center, and the number of times that individuals reported being enrolled in a drug rehabilitation center.

### Data analysis

Univariate logistic regression was used to examine associations between independent and dependent variables. Independent variables significant at an alpha level of 0.05 in univariate analyses were entered forward stepwise into a multivariate logistic regression model. Unadjusted and adjusted odds ratios at a 95 % confidence interval were calculated. All analyses were performed using SPSS 21.0 statistical software [43].

## Results

A total of 733 individuals who injected drugs in the last 30 days and resided in Tijuana, Mexico were recruited into the *Proyecto El Cuete* Phase IV cohort between March 2011 and May 2013, and provided baseline data for the current study. Participants’ median age was 37 years (Interquartile range [IQR]: 31, 44) and 38 % were female (Table 1). Almost half (45 %) of the sample reported being married or in a relationship, and the median number of years of education achieved was 8 (57 %). One-fifth of the sample was homeless (20 %) and close to one-third (28 %) reported informal employment as their main source of income. The three primary drugs participants reported ever using were heroin (100 %), crystal methamphetamine (88 %) and cocaine (76 %). Fifty-one percent of the sample expressed a great or urgent need to get help for their drug use. One third of the sample perceived risk of violence at drug rehabilitation centers as indicated by their agreement with the statement “*going to drug treatment puts me at risk for violence*” (34 %, *n* = 253).

**Table 1:** Demographic characteristics (n=733)

Variable name	Total	Feared violence	Didn't fear violence	p-value^a^
	(n=733)	(n=253, 34.5%)	(n=480, 65.5%)	
Age^b^	37 (31, 44)	36 (30, 42)	37 (31, 45)	0.08
Female (n, %)	279 (38.1%)	102 (40.3%)	177 (36.9%)	0.38
Married (n, %)	332 (45.3%)	113 (44.7%)	219 (45.6%)	0.82
Education				
Years of education completed^b^	8 (6,10)	8 (6,11)	8 (6,10)	0.15
Main source of income				
No income (n, %)	25 (3.4%)	8 (3.2%)	17 (3.5%)	0.82
Formal source of income (n, %)	502 (68.5%)	177 (70.0%)	325 (67.7%)	
Informal source of income (n, %)	203 (27.7%)	67 (26.5%)	136 (28.3%)	
Homeless (where slept most often last 6 mos)				
Homeless (n, %)	146 (19.9%)	60 (23.7%)	86 (17.9%)	0.06
Mobility				
Lived in Tijuana whole life (n, %)	278 (37.9%)	107 (42.3%)	171 (35.6%)	0.08
Years lived in Tijuana (n= 454) ^b^	21 (10, 34)	20 (10, 34)	21 (10, 33)	0.54
Drug Use History				
Age of first time use of illegal drug^b^	14 (12,16)	14 (12,16)	14 (12,16)	0.30
Age of first injected drugs^b^	19 (17,24)	20 (17,25)	19 (17,24)	0.58
Ever used heroin	730 (99.6%)	252 (99.6%)	478 (99.6%)	0.99
Ever used cocaine	559 (76.3%)	171 (67.6%)	388 (80.8%)	<0.01
Ever used crystal/methamphetamine	647 (88.3%)	206 (81.4%)	441 (91.9%)	<0.01
Interactions with Law Enforcement				
Ever stopped by law enforcement	661 (90.2%)	220 (87.0%)	441 (91.9%)	0.04
Ever been told by law enforcement that you would be required to go to drug treatment	17 (2.3%)	10 (4.0%)	7 (1.5%)	0.04
Drug Rehabilitation History				
Great or urgent need to get help for drug use (n, %)	375 (51.2%)	144 (56.9%)	231 (48.1%)	0.02
Ever received professional help for drug use^c^	417 (56.9%)	124 (49.0%)	293 (61.0%)	0.01
Lifetime # of times received professional help for drug use (n=417) ^b^	1 (0, 3)	0 (0, 3)	1 (0, 4)	0.01
Ever enrolled in drug rehabilitation center (n, %)	375 (51.2%)	109 (43.1%)	266 (55.4%)	0.37
# of times enrolled in drug rehabilitation center(n=375) ^b^	1 (0, 3)	0 (0, 2)	1 (0, 3)	0.01

^a^comparisons of means made using Student t-test, comparisons of proportions made using chi-squared test, all p-values are two-sided

^b^Median, interquartile range

^c^Ever received professional help refers to outpatient counseling services and might include drug rehabilitation services

### Univariate and multivariate analyses

Table 2 presents results of the univariate and multivariate logistic regression analyses examining the relationship between perceived risk of violence at drug rehabilitation centers and individual, interpersonal, community and societal-level factors. Following we present the variables that were significantly associated with perceived risk of violence at drug rehabilitation centers in univariate analyses.

**Table 2:** Sociodemographic and Health Factors Associated with Risk of violence in drug rehabilitation centers (*N* = 733)

			LR Model^a^	
Variable name	OR (95 % CI)	*p*-value	Adjusted OR (95 % CI)^a^	*p*-value
Demographics				
Age	0.98 (0.97, 1.00)	0.08	0.98 (0.96, 0.99)	0.03
Female (Ref: male)	1.16 (0.85, 1.58)	0.36		
Married (Ref: not married)	0.96 (0.71, 1.31)	0.80		
Education				
Years of education completed	1.04 (0.99, 1.09)	0.16		
Main source of income				
Formal source (Ref: no income)	1.16 (0.49, 2.74)	0.74		
Informal source (Ref: no income)	1.05 (0.43, 2.55)	0.92		
Housing (where slept most often last 6 months)				
Homeless	1.43 (0.98, 2.07)	0.06	3.33 (1.21, 9.20)	0.02
Mobility				
Lived in Tijuana whole life	1.32 (0.97, 1.81)	0.08	1.09 (.78, 1.53)	0.62
Years lived in Tijuana	1.00 (0.99, 1.01)	0.49		
Drug Use History				
Age of first time use of illegal drug	1.02 (0.99, 1.06)	0.26		
Age of first injected drugs	1.00 (0.98, 1.03)	0.73		
Ever used heroin	1.05 (0.10, 11.68)	0.97		
Ever used cocaine	0.49 (0.35, 0.70)	0.01	0.64 (0.43, 0.95)	0.03
Ever used crystal/methamphetamine	0.39 (0.25, 0.61)	0.01	0.48 (0.29, 0.79)	<0.01
Interactions with law enforcement				
Ever stopped by law enforcement	0.57 (0.35, 0.94)	0.03	0.72 (0.43,1.21)	0.21
Been told by law enforcement that would be required to go to rehabilitation	2.79 (1.05, 7.41)	0.04	3.61 (1.35, 9.64)	0.01
Drug Rehabilitation History				
Great or urgent need to get help for drug use	1.44 (1.06, 1.95)	0.02	1.61 (1.17, 2.22)	<0.01
Ever received professional help for drug problem	0.61 (0.45–0.83)	0.01	0.67 (0.48, 0.93)	0.02
Lifetime # of times received professional help for drug use (*n* = 417)	0.98 (0.95, 1.01)	0.14		
Ever enrolled in drug treatment	0.74 (0.38–1.44)	0.38		
# of times enrolled in drug rehabilitation center (*n* = 375)	0.98 (0.95, 1.01)	0.21		

^a^LR (Logistic Regression) Model: Dependent Variable: Fear of violence Dichotomous - Reference (No fear of violence) - METHOD = ENTER ever used cocaine, ever used crystal/methamphetamines, ever told by law enforcement that would be required to go to rehabilitation, great or urgent need to get help for drug use, ever received professional help for drug problem and age

*Individual level variables:* none of the sociodemographic variables considered were significantly associated with perceived risk of violence at drug rehabilitation centers. Of the drug use-related variables examined, ever having used cocaine (OR = 0.49, 95 % CI: 0.35 – 0.70, *p* < 0.01) or crystal methamphetamine (OR = 0.39, 95 % CI: 0.25 – 0.61, *p* < 0.01) were negatively associated with perceived risk of violence at drug rehabilitation centers. Meanwhile, having a great or urgent need to get help for drug use (OR = 1.44, 95 % CI: 1.06 – 1.95, *p* = 0.02) was positively associated with perceived risk of violence at drug rehabilitation centers.

*Interpersonal-level variables:* ever having been stopped by law enforcement (OR = 0.57, 95 % CI: 0.35 – 0.94, *p* = 0.03) was negatively associated with perceived risk of violence at drug rehabilitation centers, while ever being told by law enforcement or other officials to attend mandatory drug rehabilitation (OR = 2.79, 95 % CI: 1.05 – 7.41, *p* = 0.04) was positively associated with perceived risk of violence at drug rehabilitation centers.

*Community/societal level variables:* ever having received professional help for drug or alcohol use (OR = 0.61, 95 % CI: 0.45 – 0.83, *p* < 0.01) was negatively associated with perceived risk of violence at drug rehabilitation centers.

Variables significant at an alpha level of 0.05 were included in our final multivariate logistic regression model. As shown in Table 2, having ever used cocaine (AOR = 0.58, 95 % CI: 0.40 – 0.85, *p* = 0.01) or crystal/methamphetamine (AOR = 0.50, 95 % CI: 0.31 – 0.82, *p* < 0.01), and ever receiving professional help for drug or alcohol use (AOR = 0.66, 95 % CI: 0.48 – 0.91, *p* = 0.01) were negatively associated with perceived risk of violence at drug rehabilitation centers. Having a great or urgent need to get help for drug use (AOR = 1.57, 95 % CI: 1.14 – 2.16, *p* <0.01) and ever being told by law enforcement or other officials to attend mandatory drug rehabilitation (AOR = 3.44, 95 % CI: 1.31 – 9.04, *p* = 0.01) were positively associated with perceived risk of violence at drug rehabilitation centers.

## Discussion

One third of our sample of PWID in Tijuana, Mexico believed that attending a drug rehabilitation center placed them at risk of violence. This is of concern, since over half of the sample reported having a great or urgent need for help with their drug use, and those who expressed great or urgent need perceived greater risk of violence at drug rehabilitation centers. Accounts of violence perpetrated against drug rehabs or within drug rehabs have taken place at “ayuda mutua” programs and among people who were recovering from alcohol or heroin use [18, 21–25, 29, 32]. Minimizing the perceived risk of violence at drug rehabilitation centers may reduce barriers to entering rehabilitation, and thus may be critical to maximizing the public health impact of Mexico’s recently reformed drug policy.

Those PWID who reported ever having used cocaine or methamphetamine were less likely to perceive risk of violence at drug rehabilitation centers. It could be that those who use methamphetamine or cocaine do not see themselves as in potential danger by attending a drug rehabilitation program. It could be that those who use methamphetamine or cocaine do not see themselves as in potential danger by attending a drug rehabilitation program. Moreover, participants who had previously received professional help for drug use were less likely to perceive violence at drug rehabilitation programs. This suggests that fears about violence were not reinforced among PWID who actually received professional drug treatment services. Professionalization of service delivery and evidence-based interventions are two aspects of the recent drug policy reforms that would benefit the peer support type of drug rehabilitation programs, historically associated with instances of violence and human rights violations in Mexico [18, 21, 25, 29, 32]

Our findings have a number of implications. First, further research is needed to explore the sources of violence (both actual and perceived) at drug rehabilitation centers and the subsequent impact of this violence on PWID’s willingness to enter rehabilitation programs in México. There is a need for scientific evidence that can substantiate victims’ reports of violence. Research characterizing and evaluating the sources of violence in drug rehabilitation centers could support the development of effective strategies to reduce actual and perceived risks of violence in drug rehabilitation centers, and potentially increase treatment uptake among PWID.

We also found out that individuals who reported that law enforcement or other officials had told them they would be required to go to rehabilitation were nearly four times as likely to perceive that attending a drug rehabilitation center placed them at risk for violence. Even though this finding refers to a small subset of the sample, it is important because mandated referrals to treatment are anticipated to increase as Mexico’s drug policy reforms (Narcomenudeo) are brought to scale. As such, this subsample may provide insight into how the broad implementation of the Narcomenudeo law may impact PWID in Tijuana and elsewhere in the country. In this context, between August and September of 2010, more than 80 % of respondents to a Mexican household survey in the 32 Mexican states reported having little or no trust in transit, municipal, and state police [44] due to actions such as illegally bursting into homes, planting evidence, torture and taking people's possessions [45]. Further, by September 2011, less than 25 % of the municipal and state police had been evaluated for trustworthiness [46]. Given these data, law enforcement officials may be feared as a source of violence.

Third, implementing a registry of all organizations providing drug rehabilitation services and requiring them to implement evidence-based interventions and professionalizing their staff could help to ensure the quality and appropriateness of the care provided in drug rehabilitation centers and minimize perceptions of fear of violence among PWID. Of concern, there is currently no data indicating the total number of agencies providing drug rehabilitation services at the state or national level or the quality of the services provided. Similarly, establishing an accreditation process for all registered entities to qualify for federal and state funding may encourage drug rehabilitation centers to comply with the requirements of the Health Law and thereby potentially reduce the possibility of mistreatment of center clients and improve enrollment and retention of drug-dependent individuals in treatment. Such coordinated efforts are currently being implemented in some states like Baja California between the Federal Addictions Agency (CONADIC) and the state counterpart, the Institute of Psychiatry of Baja California (IPEBC) and could serve as role models for expanded implementation in other states.

Experts calling for effective global public health approaches for problematic illicit drug use have identified a key role for evidence-based voluntary rehabilitation programs that are monitored on a regular basis to prevent basic human rights violations, including instances of violence [1, 2, 9, 10]. While Mexico’s legislation conforms to this new approach, our findings suggest that there is a lack of operationalization of the legislation at the law-enforcement and consumer level, which compromises its effectiveness.

The present study has several limitations. First, while associations between variables of interest can be evaluated, we cannot determine the temporality of observed relationships or make causal inferences from these cross-sectional data. Being able to ascertain the cause of fear of violence or actual violence in the context of drug rehabilitation is key to be able to provide access to quality services and achieve effective drug rehabilitation [1, 2, 20]. Second, participants were recruited using a non-probabilistic, convenience sampling approach and the generalizability of findings to the broader population of PWID in Tijuana may be limited. Efforts should be carried out to include a representative sample of PWID to better characterize all aspects of the drug use and rehabilitation landscape in the region and being able to generalize study findings. Third, as a result of safety concerns and the stigmatized nature of drug use in Mexico, it is possible that participants may have underreported their perceived risk of violence at drug treatment centers. While mass media has highlighted instances of violence in Mexico during the 2008–2011 period [22, 23, 36, 47], systematic documentation of violence among PWID at drug rehabilitation centers should be attempted by other researchers. Additionally, fear of reprisal may have caused participants to underreport violence. Fourth, we only used one single dichotomous survey item to measure study participants’ perception of the risk of violence and so it offers a very limited perspective. Ideally, when examining such a complex concept of perception of risk of violence qualitative approaches together with quantitative measurements are needed to enhance measurement and understanding of the phenomenon. Finally, we did not collect data regarding the nature of the perceived violence (e.g, physical, psychological and or sexual) or who the putative perpetrator may be (e.g., law enforcement officials, other drug users, drug cartels or staff at the drug rehabilitation centers). Ongoing research undertaken by our group seeks to investigate this phenomenon.

## Conclusion

Among a cohort of PWID in Tijuana, one-third of the sample agreed that going to rehabilitation put them at risk of violence, while those who had previously received professional help for their substance use had a lower odds of fearing violence. This suggests that fears regarding the level of violence in drug rehabilitation centers were not reinforced among PWID who actually received drug treatment services delivered by professionals. Similarly, even though it represented a small percent of the total sample, individuals who had ever been told by law enforcement that they were required to enter into a drug rehabilitation center were more likely to perceive that going to rehabilitation put them at risk for violence. Our findings bolster the need to meaningfully implement the reforms to the health, penal and judicial laws that were enacted in 2009 but have not yet been effectively scaled up. This includes, in particular, the need to strengthen the infrastructure and systematic evaluation of all drug rehabilitation centers in Tijuana and the rest of the country. Professionalization of service delivery at peer support centers and regular auditing of compliance with enacted laws and regulations are paramount for the success of an effective drug rehabilitation strategy as intended. Under Mexico’s drug policy reforms, the system of diversion of drug-dependent individuals to addiction treatment relies on coordinated partnerships between health providers and law enforcement to facilitate referral and access to certified drug rehabilitation centers. Health authorities should therefore expand periodic evaluations of such centers to ensure full compliance with the health law in order to minimize barriers and encourage uptake of, and retention in, treatment. To support this effort, law enforcement should also be trained to ensure that interactions with PWID serve to facilitate enrolment into evidence-based addiction treatment services. A police education program is now being offered in Tijuana by our team, in partnership with the Tijuana police department which we hope will address this concern.

## Additional file

Additional file 1:
**STROBE Statement—checklist of items that should be included in reports of observational studies.** (DOCX 30 kb)
